# Case Report: ICIs-induced Guillain–Barré syndrome recovered from mycophenolate mofetil

**DOI:** 10.3389/fimmu.2023.1132692

**Published:** 2023-05-08

**Authors:** Mengge Ding, Chao Deng, Xianling Liu, Shun Jiang, Yuan Gao, Dan Fan, Yiguang Zhou, Jiangbo He, Chaoyuan Liu

**Affiliations:** ^1^ Department of Oncology, The Second Xiangya Hospital, Central South University, Changsha, Hunan Province, China; ^2^ Department of Biomedical Education and Data Sciences, Temple University School of Medicine, Philadelphia, PA, United States

**Keywords:** Guillain–Barré syndrome, immune checkpoint inhibitors, immune-related adverse events, KN046, mycophenolate mofetil

## Abstract

The emergence of immune checkpoint inhibitors (ICIs) has significantly prolonged the survival time of cancer patients. However, it may also lead to various immune-related adverse events (irAEs), including Guillain–Barré syndrome (GBS), a rare type of irAE. Most GBS patients can recover spontaneously due to the self-limited nature of the disease, but severe cases can result in respiratory failure or even death. Here we report a rare case of GBS occurring in a 58-year-old male patient with non-small cell lung cancer (NSCLC) who developed muscle weakness and numbness of the extremities during chemotherapy combined with KN046, a PD-L1/CTLA-4 bispecific antibody. Despite receiving methylprednisolone and γ-globulin, the patient’s symptoms did not improve. However, there was significant improvement after treatment with mycophenolate mofetil (MM) capsules, which is not a routine regimen for GBS. To the best of our knowledge, this is the first reported case of ICIs-induced GBS that responded well to mycophenolate mofetil instead of methylprednisolone or γ-globulin. Thus, it provides a new treatment option for patients with ICIs-induced GBS.

## Introduction

Immune checkpoint inhibitors (ICIs)-based immunotherapy has revolutionized the therapeutic landscape of malignant tumors in recent years. Antibodies directed against programmed cell death protein-1 (PD-1), anti-PD-1 ligand (PD-L1), and antibody targeting cytotoxic T lymphocyte-associated antigen-4 (CTLA-4) are the representatives of ICIs. ICIs-based immunotherapy has not only extended the survival time but also significantly improved the living quality of patients with certain cancers, such as melanoma, lung cancer, liver cancer, and breast cancer (1). However, due to the activation of the immune system, immune-related adverse events (irAEs) can also involve almost all the organs (2).

Guillain–Barré syndrome (GBS) is an autoimmune peripheral neuropathy characterized by demyelinating lesions of peripheral nerves and nerve roots and inflammatory cell infiltration of small vessels. The most common clinical manifestation of GBS patients is acute symmetrical flaccid limb paralysis. A recent meta-analysis found that although immune-related neurological adverse events (NAEs) are relatively rare compared to chemotherapy, they often result in high mortality and disability (3). Guillain–Barré syndrome (GBS) induced by ICIs accounted for 0.3% of all irAEs and has similar symptoms to classic GBS. Despite receiving standard treatment, many GBS patients still experience residual symptoms for an extended period, significantly affecting their quality of life (4).

ALPHAMAB ONCOLOGY has independently developed a PD-L1/CTLA-4 bispecific antibody called KN046 which is composed of a CTLA-4 with a PD-L1 single-domain antibody. KN046 aims to achieve a dual targeting effect by simultaneously targeting the tumor microenvironment, which is known to be enriched in PD-L1, and by eliminating regulatory T-cells (Tregs) that inhibit tumor immunity. This innovative approach could potentially result in better treatment outcomes for patients with cancer.

Here we report a rare case of GBS occurring in a 58-year-old male patient with non-small cell lung cancer (NSCLC) who developed muscle weakness and numbness of the extremities during treatment with chemotherapy combined with KN046. Based on the results of an electromyogram (EMG) and cerebrospinal fluid (CSF) analysis, he was diagnosed with ICIs-induced GBS. Traditional treatment of GBS including methylprednisolone and immunoglobulin (IVIG), was used to relieve his symptoms. However, these treatments were initially ineffective until mycophenolate mofetil (MM) capsules were prescribed, which resulted in a significant improvement in his symptoms. Our study discusses the diagnosis and treatment characteristics of GBS and provides a non-routine but feasible treatment option for ICIs-induced GBS patients.

## Case report

A 58-year-old male patient who presented with recurrent thoracalgia was admitted to our department. A CT scan of his lungs identified a lump in his left lobe, and a biopsy through fiberoptic bronchoscopy confirmed lung squamous cell carcinoma. The PD-L1 tumor proportion score (TPS) was 5%. After a series of examinations, including single photon emission computed tomography (SPECT) and magnetic resonance imaging (MRI) of the brain, the diagnosis of primary lung squamous cell carcinoma (cT4N3M0, IIIC, AJCC 8th) was confirmed. The patient had no previous history of other chronic diseases or family cancer history, and was a smoker for 30 years with an average of one pack per day. After screening, the patient was enrolled in a multicenter, randomized, double-blind, placebo-controlled phase III clinical trial to assess the efficacy and safety of KN046 combined with platinum-based chemotherapy compared to placebo and platinum-based chemotherapy in patients with advanced squamous non-small cell lung cancer. The clinical trial registration number is CTR20201294, and the patient’s random number is 376. In August 2021, the patient began the first cycle of treatment, which included an intravenous infusion of paclitaxel 261 mg and carboplatin 490 mg, with an intravenous infusion of KN046 or placebo 240 mg. On the third day after medication, the patient developed mild pain in his lower limbs, for which ibuprofen was prescribed to alleviate the discomfort. Subsequently, the patient was discharged.

Twenty-one days later, the patient was readmitted to the hospital in a wheelchair due to symptoms of fatigue, muscle weakness, and limb pain that had developed over the course of three days. Physical examination revealed grade 1 muscle strength in upper and lower limbs, accompanied by mild bilateral ptosis. Various markers including procalcitonin, erythrocyte sedimentation rate, C-reactive protein, and myoglobin showed varying degrees of increase. The cerebrospinal fluid (CSF) analysis showed a clear appearance, with no cells and mildly elevated glucose (6.38 mmol/L; Normal range 2.5-4.5 mmol/L), and elevated protein level (999 mg/L; Normal range 150-450 mg/L). The electromyogram (EMG) revealed slowed nerve conduction velocity and abnormal amplitude in multiple parts of the patient’s upper and lower limbs. Given the acute onset and progression of the disease, and the CSF analysis result showed a typical “albumino-cytologic dissociation” phenomenon, the patient was diagnosed with ICIs-induced GBS by the neurologist. Anti-tumor treatment was ceased and a serious adverse event (SAE) was reported. The patient was excluded from the study and unblinded; it showed that he had been assigned to the KN046 drug group.

**Figure 1:**
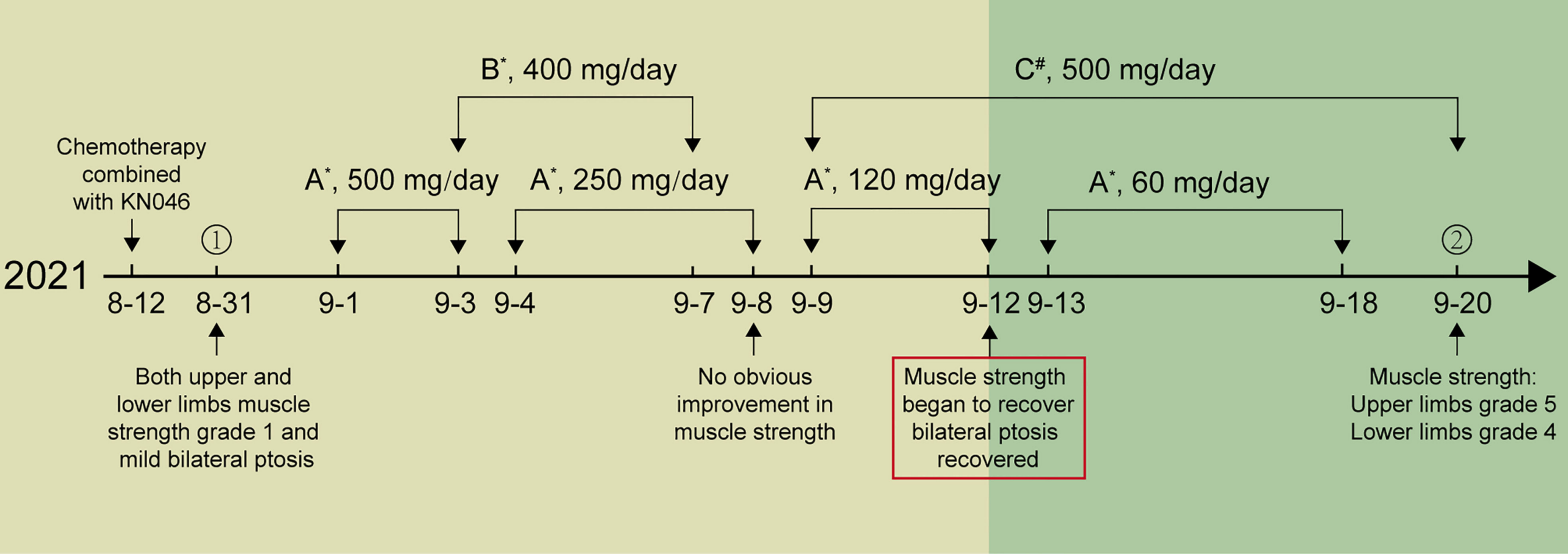
The treatment procedure of the patient. **(A)** Methylprednisolone, **(B)** IVIG, **(C)** Mycophenolate mofetil capsules; *ivgtt, ^#^po. ① Day of admission. ② Day of discharged.

Initially, the patient was treated with intravenous methylprednisolone of 500 mg and immunoglobulin (IVIG) of 20 g (0.4 g/kg) per day, in accordance with the Guidelines for the management of immune checkpoint inhibitor-associated toxicity 2021 by the Chinese Society of Clinical Oncology (CSCO). Mecobalamin and pregabalin were used together to nourish the nerves and improve the symptoms of weakness and numbness of limbs. However, there was no improvement in the patient’s myasthenia after three days of treatment. Then the patient was given mycophenolate mofetil (MM) capsules 500 mg twice a day. After taking the MM capsules for three days, the patient showed significant improvement in muscle strength, progressing from grade 1 to grade 3, and bilateral ptosis gradually recovered. Methylprednisolone was reduced gradually as the patient’s symptoms improved. After 19 days of treatment, the patient’s myodynamia recovered when he was discharged from the hospital, and the muscle strength of his upper limbs and lower limbs recovered to grade 5 and 4, respectively. The patient was able to walk out of the hospital on his own. The treatment procedure is shown in **Figure 1**. Chemotherapy and KN046 therapy were discontinued permanently for the patient and he eventually died of cancer progression on May 24, 2022.

## Discussion

The use of immune checkpoint inhibitors (ICIs) is highly effective in restoring and enhancing the cytotoxic T cells’ anti-tumor activity. However, overactivation of T lymphocytes can occur systemically and may lead to not only an anti-tumor immune response but also autoimmunity, resulting in various irAEs (5). A study focused on toxicity spectrum of immunotherapy in advanced lung cancer found that chemotherapy combined with both PD-L1 and CTLA-4 antibody was associated with the highest risk of grade 1–5 irAEs compared with ICI monotherapy and chemotherapy alone (6). Unlike other irAEs, neurological complications caused by ICIs are rare (7) and often occur in melanoma patients (8). Of note, ICIs-related neurological complications do not correlate with autoantibodies associated with these drugs. This suggests that there might be T-cell-mediated pathogenesis instead (9).

GBS is currently classified as an immune-mediated polyradiculoneuropathy, with most patients presenting neurological symptoms after an infective illness (10). It has been reported that GBS has relationships with particular vaccinations (11), ganglioside administration (12), surgery (13) and immune checkpoint inhibitors therapy. According to the site of target antigen and occurring frequency, GBS can be mainly classified into acute inflammatory demyelinating, acute motor axonal neuropathy (AMAN), and polyradiculoneuropathy (AIDP) (14). The main difference between AMAN and AIDP is whether patients have substantial T-cell inflammation or demyelination (10). An autopsy study of 11 patients who died of GBS showed that ADIP was characterized by the presence of inflammatory infiltrates containing T cells and macrophages involved in macrophage-mediated demyelination (15). Compared with AIDP, patients with AMAN always show primary axonal injury without substantial T-cell inflammation or demyelination (10). The incidence of GBS increased by 20% with every 10-year increase in age, whether in ICIs-induced GBS or other forms of GBS, and males have a higher risk of contracting GBS (16). The exact mechanism by which ICIs induced GBS is still unknown; one hypothesis suggest that the abrogation of self-tolerance may activate cytotoxic T lymphocytes with a reduced inhibition of antigen-producing B lymphocytes (10). Notably, patients with pre-existing GBS have a higher risk of recurrence and exacerbation when receiving ICIs treatment (17).

We presented a case of GBS which occurred in a 58-year-old male patient with lung cancer, who experienced muscle weakness and numbness in his extremities after the first cycle of treatment with chemotherapy combined with KN046. This patient was diagnosed with ICIs-induced GBS. Notably, antitumor chemotherapy regimens, such as platinum-based agents, taxanes, vinca alkaloids, and thalidomide analogs, can also cause direct neurotoxicity and chemotherapy-induced peripheral neuropathy (CIPN) (18). These may present similar symptoms to GBS, including paresthesia, pain, symmetrical numbness, and so on (19). In contrast, CIPN is characterized only by numbness and pain in the limbs without changes in cerebrospinal fluid analysis and decreases in muscle strength. Reported cases of GBS caused by chemotherapy are sporadic. Two reported cases of GBS induced by chemotherapy included one patient with metastatic rectal cancer who developed GBS after several cycles of chemotherapy (20), while another patient with esophageal cancer received not only chemotherapy but also radiation therapy simultaneously (21). Considering the drug usage, clinical manifestations, and inspection result of this patient, we believe that his GBS was most likely induced by KN046.

According to the CSCO 2021 guideline on ICIs-associated toxicity, corticosteroids and IVIG are traditional treatments for GBS. The combination of these treatments can improve clinical symptoms of GBS patients by 73% (22). Plasma exchange (PE) can be used as a second-line treatment if the condition worsens or if the previous treatment is ineffective. Nevertheless, one network meta-analysis suggested that PE or IVIg was more effective for GBS than corticosteroids (23). Another study reported a case of ICIs- induced GBS whose symptoms, such as limb weakness, numbness, and pain at the ends of the limbs, were significantly alleviated after high doses of intravenous gamma globulin combined with acupuncture treatments (24). In our case, the patient’s symptoms were constant after receiving the traditional treatment of GBS but improved markedly after the initiation of mycophenolate mofetil. Mycophenolate mofetil is primarily used as an immunosuppressant in patients after organ transplantation to prevent and cure organ rejection. It can also be used for autoimmune diseases or other ICIs induced irAEs but is not a routine regimen for GBS. In a previous study, the combination of IVIg-MP-MM was proven to be safe but with no efficacy for GBS patients (25). However, MM did have a positive effect in our ICIs-induced GBS. This suggest that there may be some differences between ICIs-induced GBS and other types of GBS. It is speculated that ICIs-induced GBS may be related to the decreased tolerance to ganglioside-related epitopes and an unsuppressed immune response in the periphery due to interference with the function of normal immune checkpoint molecules (26). While the specific mechanism still needs further study.

GBS is a complex neurological disease, and it takes a long time for patients to relieve from neurological symptoms. Therefore, more effective, affordable, and safer treatments for GBS are urgently needed. Our case report provides valuable insights into effective treatment options for ICIs-induced GBS.

## Conclusion

In conclusion, we report a rare case of ICIs-induced GBS who didn’t derive benefit from traditional methylprednisolone and IVIG but recovered soon after the adoption of mycophenolate mofetil. It has significant reference to similar ICIs-induced GBS.

## Data availability statement

The original contributions presented in the study are included in the article/supplementary material. Further inquiries can be directed to the corresponding author.

## Ethics statement

This study was reviewed and approved by the ethics committee of the Second Xiangya Hospital, Central South University. The patient provided his written informed consent to publish in this case report.

## Author contributions

MD: Writing-original draft. CD: Writing-review and editing. XL, SJ, YG: Validation. DF, YZ, JH: Investigation. CL: Conceptualization and methodology. All authors contributed to the article and approved the submitted version.
